# Negative effect of smoking on the performance of the QuantiFERON TB gold in tube test

**DOI:** 10.1186/1471-2334-12-379

**Published:** 2012-12-27

**Authors:** Martine G Aabye, Thomas Stig Hermansen, Morten Ruhwald, George PrayGod, Daniel Faurholt-Jepsen, Kidola Jeremiah, Maria Faurholt-Jepsen, Nyagosya Range, Henrik Friis, John Changalucha, Aase B Andersen, Pernille Ravn

**Affiliations:** 1 Clinical Research Centre, Copenhagen University Hospital Hvidovre, Kettegårds Alle 30, 2650, Hvidovre, Denmark; 2 Department of Pulmonary and Infectious Diseases, Copenhagen University Hospital Hillerød, Dyrehavevej 29, Hillerød, 3400, Denmark; 3 National Institute for Medical Research, Mwanza Medical Research Centre, Mwanza, Tanzania; 4 Department of Human Nutrition, Faculty of Life Sciences, University of Copenhagen, Büllowsvej, 1870, Frederiksberg, Denmark; 5 National Institute for Medical Research, Muhimbili Medical Research Centre, Dar es Salaam, Tanzania; 6 Department of Infectious Diseases, University Hospital, Odense, Sdr. Boulevard 29, 5000, Odense C, Denmark

**Keywords:** Tuberculosis, IGRA, HIV, Quantiferon, Smoking

## Abstract

**Background:**

False negative and indeterminate Interferon Gamma Release Assay (IGRA) results are a well documented problem. Cigarette smoking is known to increase the risk of tuberculosis (TB) and to impair Interferon-gamma (IFN-γ) responses to antigenic challenge, but the impact of smoking on IGRA performance is not known. The aim of this study was to evaluate the effect of smoking on IGRA performance in TB patients in a low and high TB prevalence setting respectively.

**Methods:**

Patients with confirmed TB from Denmark (DK, n = 34; 20 smokers) and Tanzania (TZ, n = 172; 23 smokers) were tested with the QuantiFERON-TB Gold In tube (QFT). Median IFN-γ level in smokers and non smokers were compared and smoking was analysed as a risk factor for false negative and indeterminate QFT results.

**Results:**

Smokers from both DK and TZ had lower IFN-γ antigen responses (median 0.9 vs. 4.2 IU/ml, p = 0.04 and 0.4 vs. 1.6, p < 0.01), less positive (50 vs. 86%, p = 0.03 and 48 vs. 75%, p < 0.01) and more false negative (45 vs. 0%, p < 0.01 and 26 vs. 11%, p = 0.04) QFT results. In Tanzanian patients, logistic regression analysis adjusted for sex, age, HIV and alcohol consumption showed an association of smoking with false negative (OR 17.1, CI: 3.0-99.1, p < 0.01) and indeterminate QFT results (OR 5.1, CI: 1.2-21.3, p = 0.02).

**Conclusions:**

Cigarette smoking was associated with false negative and indeterminate IGRA results in both a high and a low TB endemic setting independent of HIV status.

## Background

Diagnosis of latent tuberculosis (TB) infection continues to constitute a major problem for control of the TB epidemic. Although Interferon Gamma Release Assays (IGRAs) are more specific than the tuberculin skin test (TST), especially in BCG vaccinated individuals, IGRAs still suffer from suboptimal sensitivity [1-3]. A meta-analysis published in 2011 pooled results from 23 studies of the most widely used IGRA, the QuantiFERON-TB Gold In tube test (QFT) and found that an average of 20% of patients with active TB have a false negative or indeterminate QFT result [3].

Several explanations for the relative high rate of false negative results have been proposed, the most rational and well-documented being immuno-suppression as seen in advanced HIV infection and during treatment with immunosuppressive drugs [4-6]. In addition age < 5 years [7-9] and factors such as end stage renal disease [10] old age (>70 years) [11,12] and others have been proposed [1,7,13].

It is well documented, that smoking is an independent predictor of TB infection, progression of latent infection to active TB disease and death from TB [14,15]. A recently published study found that mice infected with *M. tuberculosis (M.tb.*) and later exposed to tobacco smoke had reduced Interferon-gamma (IFN-γ) responses following T-cell stimulation [16]. This is in accordance with other recent studies in humans showing that exposure to tobacco smoke reduces IFN-γ production in epithelial cells after antigenic challenge [17] and impairs IFN-γ mediated signaling [18-20] and vaccine efficacy [21,22]. These findings suggest a negative effect of smoking on the immune defense which in theory might affect the performance of immunodiagnostic tests, such as the IGRAs.

We hypothesized that smoking impairs IGRA performance. Thus, in this study we evaluate the effect of smoking on IFN-γ responsiveness, and the association of smoking with indeterminate and/or false negative QFT results in a cohort of Tanzanian patients and in a small Danish cohort.

## Methods

### Setting and participants

#### Denmark

Patients from three university hospitals in the Copenhagen Region diagnosed with confirmed (culture or Nucleic Acid Amplification Test) TB in 2009/2010 and having had a QFT test done up to 30 days before the TB diagnosis were identified using the national Danish TB Register. Information on patient characteristics, including smoking status, and QFT results were obtained from patient records. Exclusion criteria were: >1 week of anti-TB therapy and missing QFT or smoking status. Smokers were defined as current smokers at the time of QFT testing. Alcohol consumers were defined as those with a daily consumption of alcohol.

#### Tanzania

In 2006, 300 newly diagnosed pulmonary TB patients were recruited prospectively through the National Tuberculosis and Leprosy Program in Mwanza. Inclusion criteria were: available QFT and HIV results, available smoking history and a positive culture for *M. tb.* Exclusion criteria were: patients <15 years, pregnancy and other serious co-morbidity. Information on demographics, smoking habits and alcohol consumption were obtained by interview. Questions on smoking and alcohol habits were recorded as a simple yes/no answer. A detailed description of patient enrolment, TB diagnosis, HIV and QFT testing and sample handling is available in [13]. Sensitivity analysis of the QFT has been previously published on this material [13,23].

### Laboratory tests

#### Denmark

All QFT tests were done as part of the diagnostic work-up. At the hospital laboratory, QFT tubes were incubated, centrifuged and refrigerated. Within one day they were transported to the Staten Serum Institute where QFT ELISAs (Cellestis, Qiagen, Düsseldorf, Germany) were performed and results interpreted according to manufacturer’s instruction.

#### Tanzania

The QFT tests were taken as part of a research project [13,23]. Following stimulation, QFT plasma supernatants were frozen at −80°C and shipped to Denmark on dry ice. At Hvidovre Hospital plasma samples were thawn, QFT ELISAs were performed and results interpreted in accordance with manufacturer’s instructions.

The QFT test includes blood sample tubes coated with saline (nil), phytohaemagglutinin (PHA; mitogen) and peptides from the proteins ESAT-6, CFP-10 and TB7.7 (TB antigens) respectively. Nil levels were subtracted from IFN-γ levels in the antigen and mitogen tubes respectively. Crude IFN-γ levels were not corrected for nil levels. IFN-γ responses >10 IU/ml overshoot the upper limit of the QFT assay and were therefore set to 10 IU/ml in accordance with manufacturer’s guidelines.

### Statistical analysis

Data were analysed using SAS 9.2 (SAS Institute, Cary, North Carolina, USA). Paired proportions were compared using Chi-square or Fisher’s exact tests. Age was analysed using Wilcoxon signed-rank test. Logistic regression was used to determine odds ratios (ORs) and using the Haldane correction if cells contained zero. A model adjusted for sex, age, HIV infection and alcohol consumption was used to determine ORs, but only in Tanzanian patients due to the few patients in the Danish group. All tests were two-sided, results with a p-value ≤0.05 were considered significant.

### Ethical considerations

Ethical permission was obtained from the ethics committee of the National Institute for Medical Research (NIMR) in Tanzania. Study approval was given by The Danish Central Medical Ethics Committee. Written informed consent was obtained from all Tanzanian patients enrolled in the study. Data from Danish patients were drawn from register based data and collected in agreement and accordance with the terms of the Danish Data Protection Agency. Written consent was not required and therefore not obtained.

## Results

### Danish patients

A total of 52 patients were identified with positive culture and/or PCR for TB. Eighteen patients were excluded due to lack of information on smoking status (n = 13), >1 month between QFT result and TB diagnosis (n = 2) and age below 15 years (n = 3) respectively leaving 34 patients available for final analysis; 4 (8%) were HIV-positive. Twenty (59%) were current smokers. More smokers than non-smokers consumed alcohol (p = 0.03), but there was no difference in age (p = 0.15), sex (p = 0.10) and HIV-status (p = 0.63) (Table 1). Patients were primarily from Greenland (n = 8), Denmark (n = 7), Pakistan (n = 5), East Africa (n = 5) and Southeast Asia (n = 5). Most patients had pulmonary TB (n = 23), 8 had extrapulmonary TB (of these 4 in lymph nodes, 2 meningitis, 1 peritoneal and 1 in bone), 2 had miliary and 1 pleural TB. 

**Table 1:** Background characteristics of Danish and Tanzanian TB patients

	**Danish patients (n = 34)**			**Tanzanian patients (n = 172)**		
	**Non-smokers (n = 14)**	**Smokers (n = 20)**	**p**	**Non-smokers (n = 149)**	**Smokers (n = 23)**	**p**
**Male sex, n (%)**	8 (57)	16 (80)	0.15	89 (60)	20 (87)	0.01
**Age, median (range)**	29 (24–76)	50 (23–62)	0.10	32 (15–84)	32 (22–69)	0.50
Alcohol, n (%)*	0 (0)	7 (35)	0.03	27(18)	16 (70)	<0.001
**HIV, n (%)**	1 (7)	3 (15)	0.63	71 (48)	4 (17)	0.01

Demographic characteristics of Danish and Tanzanian study participants. P-values are for comparison between non-smokers and smokers (Wilcoxon signed rank test and chi-square test). *For Danish patients, alcohol is defined as daily consumption. For Tanzanian patients, alcohol is defined as simply reporting to consuming alcohol at any level.

### Tanzanian patients

A total of 172 patients with active culture confirmed pulmonary TB and a QFT test were included for analysis; 75 (44%) were HIV-positive (Table 1). Twenty-three (13%) were current smokers and all were cigarette smokers. More smokers were male and alcohol consumers, but fewer were HIV-infected. No difference was found in age.

### Effect of smoking on crude IFN-γ levels and QFT test performance and association with risk factors in Danish patients

**Figure 1:**
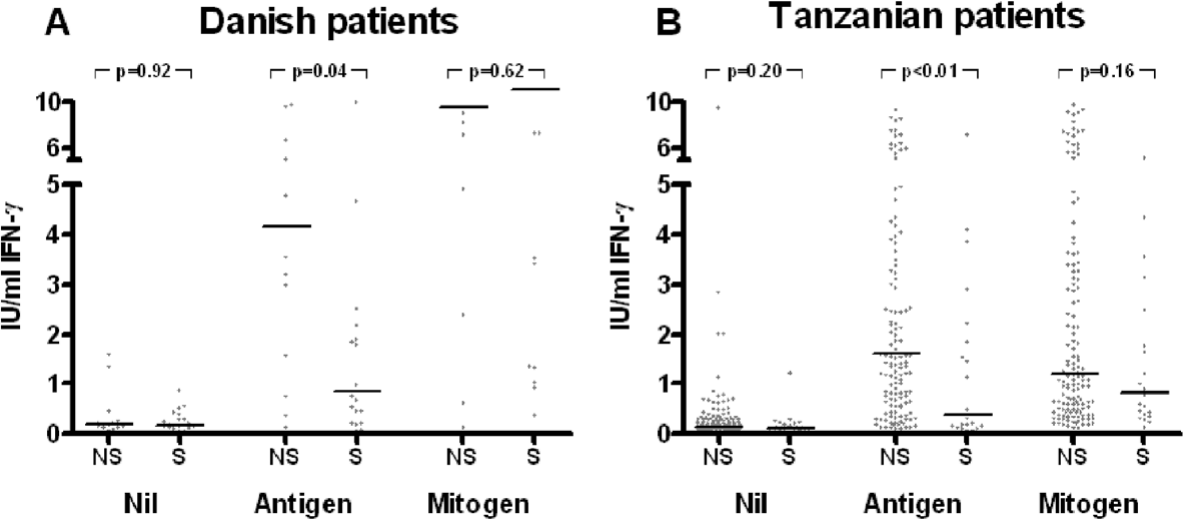
**Influence of smoking on crude IFN-γ responses in Danish and Tanzanian TB patients. **In non-smokers (NS) and smokers (S), levels of Interferon-γ were measured in supernatants from QuantiFERON-TB Gold In tube blood collection tubes after incubation with saline (Nil), *M. tuberculosis-*specific antigens (Antigen) or phytohaemagglutinin (Mitogen). Nil levels of antigen and mitogen values have NOT been subtracted. Medians and p-values for comparison of nil, antigen and mitogen levels between non-smokers (14 Danish patients and 149 Tanzanian patients) and smokers (20 Danish patients and 23 Tanzanian patients) are shown (Wilcoxon signed rank test).

Smokers had lower levels of antigen dependent IFN-γ (median 0.9 vs. 4.2 IU/ml, p = 0.04). Levels of mitogen induced IFN-γ were similar (median 9.6 vs. >10.0 IU/ml, p = 0.98). No significant differences were found in nil IFN-γ levels (median 0.19 vs. 0.18 IU/ml, p = 0.20) (Figure 1A). 

Smokers had fewer positive QFT results 50% (10/20) compared to non smokers 86% (12/14) (p = 0.03) and smokers had more false negative 45% (9/20) compared to non smokers 0% (0/14) (p = 0.004), while no difference was seen for the proportion of indeterminate results (Table 2). 

**Table 2:** Distribution of QuantiFERON-TB Gold In tube test results in non-smokers and smokers respectively in Danish and Tanzanian TB patients

	**Danish patients (n = 34)**			**Tanzanian patients (n = 172)**		
	**Non-smokers (n = 14)**	**Smokers (n = 20)**	**p**	**Non-smokers (n = 150)**	**Smokers (n = 22)**	**p**
**Positive, n (%)**	12 (86)	10 (50)	0.03	112 (75)	11 (48)	0.01
**Negative, n (%)**	0 (0)	9 (45)	0.004	16 (11)	6 (26)	0.04
**Indeterminate, n (%)**	2 (14)	1 (5)	0.56	21 (14)	6 (26)	0.14

Distribution of QFT and IP-10 results for non-smokers and smokers respectively compared by chi square test. Each outcome was tested against the pooled proportion of the other possible outcomes. QFT: QuantiFERON-TB Gold In tube test.

By univariate analysis comparing false negative and indeterminate results respectively to positive results, smoking was associated with a false negative (OR 11.3, 95% CI: 1.2-287.7, p = 0.02), but not with an indeterminate QFT result (OR 0.6, 95% CI: 0.05-7.6, p = 0.69). All (9/9) patients with false negative results were either smokers or HIV-infected. Logistic regression analysis was not performed in Danish patients due to low n and lack of power.

### Effect of smoking on crude IFN-γ levels and QFT test performance and association with risk factors in Tanzanian patients

Smokers had lower levels of antigen dependent IFN-γ (median 0.39 vs. 1.60 UI/ml, p < 0.01). There was no significant reduction in levels of mitogen induced IFN-γ in smokers (median 0.83 vs. 1.21 IU/ml, p = 0.16). No significant differences were found in nil levels (Figure 1B).

Smokers had fewer positive QFT results 48% (11/22) compared to non-smokers 75% (112/150) (p = 0.01) and more a false negative QFT 26% (6/22) versus 16% (11/150)(p = 0.04) (Table 2). No significant difference was found in the proportion of indeterminate results.

By univariate analysis comparing false negative and indeterminate results respectively to positive results, smoking was associated with a false negative (OR 3.8, 95% CI: 1.2-11.8, p = 0.02), but not with an indeterminate QFT result (OR 2.9, 95% CI: 0.97-8.7, p = 0.06) (Table 3). 

**Table 3:** Odds ratios for the association between smoking and false negative or indeterminate result in an univariate analysis for 34 Danish and 172 Tanzanian TB patients

	**False negative QFT**		**Indeterminate QFT**	
	**OR (95% CI)**	**p**	**OR (95% CI)**	**p**
**Danish TB pt**	11.3, 95% CI: 1.2-287.7	p = 0.02	0.6, 95% CI: 0.05-7.6	p = 0.69
**Tanzanian**	3.8, 95% CI: 1.2-11.8	p = 0.02	2.9, 95% CI: 0.97-8.7	p = 0.06

Odds ratios (OR) were for false negative results and indeterminate results in univariate analysis in 34 Danish and 172 Tanzanian patients.

Possible predictors for a false negative and indeterminate QFT result respectively were identified by univariate analysis in 34 Danish and 172 Tanzanian patients with bacteriologically confirmed pulmonary TB (Table 3).

Logistic regression analysis adjusted for sex, age, HIV infection and alcohol consumption (Table 4) showed an association between smoking and false negative results (OR: 17.14 (95%CI: 2.96-99.11, p = 0.0015) and indeterminate results (OR: 5.13 (1.24-21.25, p = 0.02). There was an association between HIV and indeterminate results (OR: 4.34 1.59-11.85, p = 0.0041), but not false negative results (OR: 2.11 (0.73-6.14, p = 0.17). We found an inverse association with alcohol consumption and false negative results (OR: 0.12(0.02-0.77))(p = 0.03), but not with indeterminate results (0.60, (0.18-1.97)) (p = 0.40). 

**Table 4:** Odds ratios for indeterminate or false negative result by multivariate logistic regression analysis for 172 Tanzanian patients only

	**False negative QFT**		**Indeterminate QFT**	
	**OR (95% CI)**	**p**	**OR (95% CI)**	**p**
**Sex (male)**	0.99 (0.34-2.90)	0.99	2.56 (0.88-7.51)	0.09
**Age**	0.99 (0.95-1.03)	0.60	0.98 (0.95-1.02)	0.37
**HIV**	2.11 (0.73-6.14)	0.17	4.34 (1.59-11.85)	0.0041
**Smoking**	17.14 (2.96-99.11)	0.0015	5.13 (1.24-21.25)	0.02
**Alcohol consumption**	0.12 (0.02-0.77)	0.03	0.60 (0.18-1.97)	0.40

Possible predictorsfor a false negative and indeterminate QFT result respectively were identified by multivariate analysis in 172 patients with bacteriologically confirmed pulmonary TB from Tanzania in a model including sex, HIV, smoking and alcohol consumption.

QFT: QuantiFERON-TB Gold In tube test. OR: Odds ratio. CI: Confidence interval.

Patients who had smoked for more than five years had more false negative or indeterminate results than patients who had smoked for less than five years, but the difference was not statistically significant (58 vs. 25%, p = 0.57) (data not shown).

## Discussion

This is, to our knowledge, the first publication showing that IGRA performance is negatively affected by smoking. We have shown that QFT performance is impaired in TB patients that are cigarette smokers in both a high and a low TB prevalence setting. Smokers had lower crude IFN-γ responses to the TB specific antigens and smoking was associated with both false negative and indeterminate results QFT results. Since the introduction of IGRA, we and others, have been able to identify several risk factors associated with impaired performance where the IGRA results should be interpreted with caution [4,13]. The present study adds further information to this knowledge and should help us to interpret indeterminate results, explain false negative results, and in the end led to rational clinical use of the IGRAs.

Our results are in line with several previous publications showing that T cell function is greatly reduced in smokers [16-20,24,25]. This immunological impairment in smokers has been shown to be of notable clinical importance, e.g. by increasing incidence, morbidity and mortality of infectious diseases [26] as well as impairing vaccine efficacy, even in children exposed to second hand smoking [21,22].

Our findings suggest that in both high and low resource settings, caution might be necessary when interpreting IGRA results in smokers, especially since smoking was strongly associated with false negative and not just indeterminate results. Although becoming less common in most high resource settings where implementation of IGRAs is at present considered most realistic, smoking is still prevalent in many high resource countries, especially among the sub populations that also have the highest risk of TB infection. Furthermore, smoking is becoming increasingly common in low resource TB/HIV endemic areas [14]. Smoking increases morbidity and mortality from TB disease [14,15,27-29], suggesting that the increasing smoking prevalence in high burden regions could add to the effects of HIV in fuelling the TB epidemic. Further adding to this possibly devastating effect of smoking, the prospect of a potential benefit from using IGRAs as part of the TB control strategy in low resource settings seem, if possible, even more bleak.

Smoking was associated with more indeterminate QFT results in the Tanzanian patients in our study also when adjusting for HIV infection. This is in line with previous studies showing that IFN-γ responses to mitogen stimulation are impaired in smokers compared to non-smokers [16-20,24,25]. We were therefore surprised to find that smokers did not have lower median IFN-γ responses to the mitogen in the present study. The lack of a difference in mitogen responses between smokers and non-smokers might be explained by a lack of power or by the fact that the mitogen responses frequently overshot the upper limit of the QFT assay making quantification of responses in the high range impossible. Another explanation is that smokers have a higher white blood cell count and previous studies have suggested that this is a compensatory mechanism for T cell anergy [24]; A higher white blood cell count might thus recuperate at least some of the IFN-γ response to mitogens.

We found that more smokers consumed alcohol, but this seemed to have a protective effect against having a false negative QFT result. Alcohol consumption has previously been identified as a protective factor for all-cause mortality in epidemiological studies and the effects is believed to be driven by the fact that patients who are very sick might be less inclined to drink alcohol [30]. However, severely ill patients might be more likely to have an indeterminate or false negative QFT result [31]. Thus the apparent protective effect of alcohol consumption remains unanswered.

### Limitations

We used active TB infection as a golden standard for infection with *M.tb* and as a proxy for LTBI in order to determine the effect of smoking on antigen specific responses. IGRA are not recommended for diagnosing active TB, and using active TB as proxy for LTBI is controversial, but necessary in hypothesis generating studies as the present.

The immune response in patients with active TB may be compromised due to ongoing infection and the consequence may be biased results if. i.e. smokers with active TB were more severely ill compared to non-smokers. Using active TB as a surrogate for LTBI is still the only way to screen potential of risk factors influencing IGRA test performance and similar approach has been used to evaluate the performance of IGRAs in HIV co-infected TB patients. A strategy that resulted in recommendations against the use of IGRAs in patients with compromised immune function [32].

Our results cannot be directly applied to healthy persons with LTBI, and studies in healthy individuals are needed to confirm our results.

The numbers of smokers in the studies were relatively small and because of missing data we did not have sufficient power to determine the impact of the number of cigarettes smoked. Also due to low sample size, the logistic regression model could not be expanded to the Danish cohort and to include other possible predictors, such as co-morbidities or effect of immunosuppressive treatment.

## Conclusion

In conclusion, this study suggests that smoking is an independent predictor for false negative and indeterminate IGRA results in patients with active TB. This study adds further information on the limitations of IGRA, which may help us to interpret indeterminate results, explain false negative results, and in the end led to rational clinical use of the IGRAs.

## Competing interests

Copenhagen University Hospitals, Hvidovre holds pending and issued patents disclosing the use of IP-10 as a diagnostic marker for infection with *M.tuberculosis*, Martin Aabye, Pernille Ravn and Morten Ruhwald are registered as inventors.

Parts of the results from this study have been presented at Oral session at the European Respiratory society Annual Meeting Vienna, Austria in September 2011, and at the and at the 3rd Global Symposium on IGRAs in Hawaii, USA in January 2012.

Apart from above we do not have any financial or non -financial competing interests.

## Authors’ contributions

Conception and design: TSH, HF, NR, JC, ABA, PR, MR, MGA; Acquisition of data: TSH, GP, KJ, MFJ, DFJ, MR, MGA; Analysis and interpretation: PR, MR, MGA; First draft written by: MGA. All authors read and approved the final manuscript.

## Pre-publication history

The pre-publication history for this paper can be accessed here:

http://www.biomedcentral.com/1471-2334/12/379/prepub
